# A randomized study protocol comparing the platelet-rich plasma with hyaluronic acid in the treatment of symptomatic knee osteoarthritis

**DOI:** 10.1097/MD.0000000000023881

**Published:** 2021-01-15

**Authors:** Deyan Li, Yu Wang, Yi Shen

**Affiliations:** Department of Neurorehabilitation, Anhui Wannan Rehabilitation Hospital, Anhui, China.

**Keywords:** Platelet rich plasma, knee osteoarthritis, hyaluronic acid, pain, prospective, protocol

## Abstract

**Background:**

In recent years, intra articular injection of platelet rich plasma has attracted increasing attention. The major aim of our current randomized controlled double-blind study was to compare long-term outcomes of intra-articular injection of hyaluronic acid or platelet rich plasma in the treatment of the patients with knee osteoarthritis.

**Methods:**

This is a kind of double-blind, randomized, prospective, and comparative clinical investigation with the allocation ratio of 1:1 and was approved by our institutional review Committee. Between 2020 and 2021, altogether 2 hundred patients will be selected to participate in our present study. We will report the randomized experiments in accordance with the guidelines of Consolidated Standards of Reporting Trials and then offer the Consolidated Standards of Reporting Trials flow chart. The inclusion criteria were: patients aged from 40 to 70 years old, patients with chief complaint history of at least 1 month and knee joint pain for nearly 6 months, need the analgesic drug treatment, and radiology confirmed knee osteoarthritis. The eligible patients would be randomly divided into 2 groups through applying the random numbers generated by computer before surgery. Outcomes after treatment were assessed using the Western Ontario and McMaster University and the scoring systems of visual analogue scale which were recorded through questionnaires accomplished via the patients prior to the first injection and then at three and six months, 1 and 2 years follow-up. Any adverse events occurred within 1 year after surgery were recorded during follow-up.

**Results:**

This should suggest whether biological methods can offer more lasting outcomes than the viscosification.

**Trial registration:**

This study protocol was registered in Research Registry (researchregistry6265).

## 1 Introduction

Knee osteoarthritis is 1 of the most prevalent causes of loss of function, disability and debilitating pain in adults. As a result, knee osteoarthritis imposes a huge social and economic burden, costing more than $460 billion a year in lost wages and treatment costs.^[1,2]^ As a progressive and complex disease involving metabolic, genetic and biomechanical factors, there is currently no treatment for osteoarthritis. Although knee replacement provides an effective solution for severe osteoarthritis in the elderly population, concerns about implant longevity and revision surgery in younger and middle-aged patients with less severe osteoarthritis require attention to conservative approaches (for instance, steroids, hyaluronic acid, and non-steroidal anti-inflammatory drugs) to symptomatic remission and maintenance of function.^[3–5]^

Hyaluronic acid is widely used in these conservative treatments. The advantage of hyaluronic acid is that it can achieve short-term pain relief without affecting the natural course of osteoarthritis of the knee, although multiple injections may increase the patient's financial burden.^[6]^ Some studies suggest that multiple injections of hyaluronic acid during future joint replacements may increase the risk of infection.^[7–9]^ Therefore, hyaluronic acid cannot meet the requirements for effective treatment of osteoarthritis. In recent years, intra articular injection of platelet rich plasma (PRP) has attracted increasing attention. PRP is the platelet concentrate extracted via centrifugation from the whole blood. It includes a lot of growth factors and protein, containing transformed growth factor β and platelet-derived factors. It is considered to have many significant physiological functions, for instance, anti-inflammatory, analgesic, chondrocyte proliferation, and cartilage repair.^[8,10,11]^

Most previous studies have focused on the advantages and disadvantages of hyaluronic acid and PRP in treating the knee osteoarthritis. Nevertheless, most of the studies pay attention to the short-term evaluations of PRP, resulting in a lack of the data on the long-term results, this may be the major aspect of determining the superiority of 1 treatment over another.^[12–16]^ Despite the short-term follow-up (usually six months to twelve months after the injection) did not show significant advantage, longer evaluations may show differences in the duration of clinical benefit between treatments. The major aim of our current randomized controlled double-blind study was to compare long-term outcomes of intra-articular injection of hyaluronic acid or PRP in the treatment of the patients with knee osteoarthritis. This should suggest whether biological methods can offer more lasting outcomes than the viscosification.

## 2 Materials and methods

This is a kind of double-blind, randomized, prospective, and comparative clinical investigation with the allocation ratio of 1:1 and was approved by institutional review Committee in Anhui Wannan Rehabilitation Hospital. Between 2020 and 2021, altogether two-hundred patients will be selected to participate in our present study (registered at Research Registry: researchregistry6265). We will report the randomized experiments in accordance with the guidelines of Consolidated Standards of Reporting Trials and then offer the Consolidated Standards of Reporting Trials flow chart.

### 2.1 Patients

The inclusion criteria were: patients aged from 40 to 70 years old, patients with chief complaint history of at least 1 month and knee joint pain for nearly 6 months, need the analgesic drug treatment, and radiology confirmed knee osteoarthritis. Exclusion criteria were combination of systemic disease, malignant tumor, the history of platelet and autoimmune diseases, body mass index greater than 33 kg/m^2^, the use of antiplatelet medications or anticoagulant medications in the former 10 days or the aspirin in the previous 7 days or non-steroidal anti-inflammatory drugs in the last 2 days before the injection, patients with a history of the systemic steroids 2 weeks before injection, the active ulcer of knee, the septic arthritis of knee, the recent history of serious knee trauma, keen varum or valgum more than 20°, allergic to hyaluronic acid or the proteins of chicken and egg.

### 2.2 Randomization and blinding

The eligible patients would be randomly divided into 2 groups through applying the random numbers generated by computer before surgery. Researchers were not involved in intervention, the evaluation of outcome, statistical analysis or the collection of data. Random numbers were kept in the opaque sealed envelopes, all the patients were asked to chose a random envelope for the determination of treatment group. All the data collectors, surgeons, statistical analysts, as well as result assessors were not aware of grouping assignment.

### 2.3 PRP preparation

PRP was prepared by extracting the venous blood (approximately 40 mL) via applying 18-gauge needle from anterior cubital vein. Afterwards, 5 mL of citric acid gluconic acid solution was added and utilized as an anticoagulant. And then, blood samples could be centrifuged for fifteen minutes at 1500 RPM to generate 2 distinct layers, containing the plasma (upper layer) and the deposits of red blood cells (lower the layer). After the separation of plasma, it was centrifuged for 7 minutes at 3500 RPM to generate 2 novel layers in which the platelets in the lower layer of white precipitate. After removing upper layer, through shaking, the residual 4 to 6 mL can mix with white platelet precipitate. The final product is PRP (4 to 6 mL).

### 2.4 Intervention protocol

All intra-articular injections were administered by the first author using a anteromedial approach in an compartment designed to prevent the patient from knowing the patient's identity and to blind the patient to the type of injection received. Each of patient was asked to sit in the chair with their knees bent 90 degrees and the patient was separated from syringe through the opaque curtain. Meanwhile, a lot of subjects may be given injections of distinct materials in bilateral knee joints. Our goal is to make the sensation of joint filling in each knee joint as vague as possible to keep blindness. Therefore, 2 mL of specified therapeutic material was uniformly given in each injection. During injection, the local anesthesia was not utilized to avoid the side effects on the activation of platelet through changing the pH value of environment. All patients in both groups received additional acupuncture treatment.

### 2.5 Outcome measures and follow-up

Outcomes after treatment were assessed using the Western Ontario and McMaster University and the scoring systems of visual analogue scale which were recorded through questionnaires accomplished via the patients prior to the first injection and then at 3 and 6 months, 1 and 2 years follow-up. Any adverse events occurred within 1 year after surgery were recorded during follow-up. All the clinical assignments were implemented through an independent physician who was not participated in the process of injection. In the event of symptoms recurrence and adverse event, each of the patient was required to contact their attending physician with the telephone for the determination of the duration of beneficial effects offered through intra-articular injection. The patient remained double-blind until the 1-year evaluation. After that, their further assessment was unblinded.

### 2.6 Statistical analysis

SPSS version 25.0 was applied for the analyses. Continuous variables are expressed as the mean ± SDs, mean with a 95% CI or median as appropriate. The categorical variables could be reflected as the percentages and numbers. Between the groups, the comparison of continuous variables could be implemented with Mann- Whitney *U* test and Student *t*- test. Furthermore, the analysis of categorical data were carried out with Fisher exact test or χ 2 test. Taking into account the possibility of missing data, the design of the repeated measurement researches, the heterogeneity of differences in different circumstances (namely, over time), as well as the intra-individual correlation error, we utilized the generalized estimation equation to xamine the differences between Western Ontario and McMaster University and visual analog scale scores. When *P* < .05 (95 percent confidence interval excluded zero), the efficacy was viewed to be statistically significant.

## 3 Discussion

Osteoarthritis is a kind of debilitating disease that, in some way, affects as many as 47 million Americans a year, with an estimated 67 million by 2030. The increase of the incidence rate of osteoarthritis was in accordance with an increase in patients’ expectations for sustained remission of symptoms and recovery to expected activity levels. In recent years, more and more people have begun to use autologous blood products to provide both humoral mediators (the growth factors of blood) and the cellular for the healing of tissue. PRP is a kind of blood product, which offers a minimally invasive, low-cost, simple approach to acquire the growth factors concentrations. Most previous studies have focused on the advantages and disadvantages of hyaluronic acid and PRP in treating the knee osteoarthritis. Nevertheless, most of the experiments pay attention to the short-term evaluations of PRP, resulting in a lack of the data on the long-term results, this may be the major aspect of determining the superiority of 1 treatment over another. The major aim of our current randomized controlled double-blind experiment was to compare long-term outcomes of intra-articular injection of hyaluronic acid or PRP in the treatment of the patients with knee osteoarthritis.

## Author contributions

**Conceptualization:** Yu Wang.

**Data curation:** Deyan Li, Yu Wang.

**Formal analysis:** Deyan Li, Yu Wang.

**Funding acquisition:** Zhongxiao Cong.

**Investigation:** Deyan Li, Yu Wang.

**Methodology:** Yu Wang.

**Resources:** Deyan Li.

**Software:** Lejun Zhang.

**Supervision:** Yi Shen.

**Validation:** Yu Wang.

**Visualization:** Yi Shen.

**Writing – original draft:** Deyan Li.

**Writing – review & editing:** Yi Shen, Yu Wang.

## Abbreviations

Abbreviation: PRP = platelet rich plasma.
